# Decoding glioblastoma survival: unraveling the prognostic potential of olfactory function in a prospective observational study

**DOI:** 10.1186/s42466-025-00410-2

**Published:** 2025-07-24

**Authors:** Christoph Oster, Aylin Matyar, Teresa Schmidt, Thomas Hummel, Elke Hattingen, Martha Jokisch, Daniel Jokisch, Jana Grieger, Giorgio Cappello, Kathrin Kizina, Lazaros Lazaridis, Yahya Ahmadipour, Laurèl Rauschenbach, Martin Stuschke, Christoph Pöttgen, Nika Guberina, Tobias Tertel, Bernd Giebel, Gian Luca Dreizner, Francesco Barbato, Eva-Maria Skoda, Björn Scheffler, Michael Müther, Ken Herrmann, Christoph Kleinschnitz, Ulrich Sure, Cornelius Deuschl, Martin Glas, Sied Kebir

**Affiliations:** 1 Department of Neurology and Center for Translational Neuro- and Behavioral Sciences (C-TNBS), Division of Clinical Neuro-Oncology, University Medicine Essen, University Duisburg-Essen, Hufelandstr. 55, 45147 Essen, Germany; 2 German Cancer Consortium (DKTK), partner site Essen/Düsseldorf, a partnership between Deutsches Krebsforschungszentrum (DKFZ) and University Hospital Essen, Germany, DKFZ-Division Translational Neurooncology at the West German Cancer Center (WTZ), University Medicine Essen, University Duisburg-Essen, Essen, Germany; 3 German Cancer Research Center (DKFZ), Heidelberg, Germany; 4 Department of Otorhinolaryngology, Smell and Taste Clinic, Technische Universität Dresden, Dresden, Germany; 5 Department of Neuroradiology, Goethe University Hospital, Frankfurt am Main, Germany; 6 Department of Neurology, University Hospital Knappschaftskrankenhaus Bochum, Ruhr University Bochum, Bochum, Germany; 7 Department of Neurosurgery and Spine Surgery, Center for Translational Neuro- and Behavioral Sciences (C-TNBS), University Medicine Essen, University Duisburg-Essen, Essen, Germany; 8 Department of Radiotherapy, University Medicine Essen, University Duisburg-Essen, Essen, Germany; 9 Institute for Transfusion Medicine, University Hospital Essen, University of Duisburg-Essen, Essen, Germany; 10 Department of Neurosurgery, University Hospital Münster, Münster, Germany; 11 Department of Nuclear Medicine, University of Duisburg-Essen, and German Cancer Consortium (DKTK)-University Hospital Essen, Essen, Germany; 12 National Center for Tumor Diseases (NCT), NCT West, Essen, Germany; 13 LVR University Clinic for Psychosomatic Medicine and Psychotherapy Essen, University Hospital Essen, University of Duisburg-Essen, Essen, Germany; 14 Institute for Diagnostic and Interventional Radiology and Neuroradiology, University Hospital Essen, University of Duisburg-Essen, Essen, Germany; 15 Department of Neurology and Neurooncology, St. Marien Hospital, Lünen, Germany; 16 Department of Neurooncology, Center for Neurology, University Hospital Bonn, Bonn, Germany

**Keywords:** Olfactory dysfunction, Glioblastoma, Prognosis, Threshold test, Identification test

## Abstract

**Introduction:**

Olfactory impairment is common in glioblastoma and has been associated with unfavorable overall survival. However, prior studies were limited by imbalances in key prognostic factors and the absence of longitudinal olfactory assessments to evaluate treatment-related neurotoxicity. The aim of the study is to determine whether olfactory function serves as an independent prognostic marker for survival, neurocognitive outcomes, and quality of life in glioblastoma.

**Methods:**

Prospective, multicenter cohort study enrolling 64 glioblastoma patients and 64 matched healthy controls. Patients are stratified by extent of resection, O6-Methylguanine-DNA Methyltransferase promoter methylation, radiographic involvement of olfactory regions, baseline olfactory status, age, and Karnofsky performance status. Olfactory function is assessed serially using Sniffin’ Sticks (identification and threshold tests) from diagnosis through treatment. Coronal T2- and T1-weighted MRI scans are reviewed independently by two blinded neuroradiologists to detect olfactory region involvement. Neurocognitive testing, psychosocial screening, and quality of life assessments are conducted at defined intervals. Next-generation sequencing from tumor tissue is employed to explore molecular underpinnings of hyposmia. Blood samples are collected in every study visit for potential parallel translational studies.

**Perspective:**

This is the first longitudinal study evaluating olfactory function as a prognostic biomarker in glioblastoma. Findings may inform risk stratification, guide neuroprotective strategies, and improve survivorship care.

**Trial registration:**

ClinicalTrials.gov, NCT06954636, date of registration 04-16-2025 (retrospectively registered); https://clinicaltrials.gov/study/NCT06954636?cond=glioblastoma&intr=olfactory&rank=1.

## Introduction

Glioblastoma is the most common malignant brain tumor in humans, the median overall survival is between 12 and 48 months [9, 18]. Tumor therapy primarily involves maximum safe surgical resection, radiotherapy, chemotherapy, and Tumor Treating Fields. Olfactory dysfunction is widely acknowledged as a symptom of various neurological disorders, including Parkinson's disease and Alzheimer's disease [19]. The significance of olfactory function in glioblastoma remains poorly understood. Possible explanations for olfactory dysfunction in glioblastoma include microscopic infiltration of the olfactory centers by tumor cells, tumor cell migration from the subventricular zone (SVZ) via the rostral migratory pathway (RMS) toward the olfactory bulbs, the SVZ itself as a source of cellular supply during tumor development, and the side effects of tumor-specific therapies [5–7, 12, 15].

In a previous study, we demonstrated that patients with glioblastoma are significantly more prone to olfactory dysfunction than a control group of patients with non-tumorous neurological diseases not primarily associated with smell impairment [12]. The study demonstrated the prognostic relevance of an olfactory dysfunction in glioblastoma patients regardless of the olfactory tract affection by the tumor. We studied 122 patients (73 glioblastoma patients and 49 controls). All were tested at various time points using the Sniffin Sticks screening test—a 12-odor test where participants identify each odor from four predefined options. Normosmia was defined as a Sniffin Sticks score (SSC) of 9.9 or higher [10]. Patients scoring below 10 were classified as the hyposmia group (HG), while those scoring 10 or higher were classified as the normosmia group (NG) [12]. After the initial examination, 34 glioblastoma patients were assigned to the HG and 39 to the NG. O6-Methylguanine-DNA Methyltransferase (MGMT) promoter methylation, age, sex, Karnofsky performance status (KPS), tumor histology, and extent of resection did not differ significantly between the two groups. Glioblastoma patients scored significantly lower on the smell test than controls (mean SSC 8.9 vs. 9.9, *p* = 0.003). Even patients tested before chemotherapy or radiotherapy onset performed worse than controls (mean SSC 8.2 vs. 9.9, *p* = 0.001). MRI was used to determine if tumor growth affected primary olfactory centers (ethmoid bone, olfactory groove, or temporomesial structures like the posterior orbitofrontal cortex, uncus, anterior parahippocampal gyrus, entorhinal area, insula, or amygdala). The median overall survival (OS) of the cohort was 28.1 months, and the median progression-free survival (PFS) was 14 months. Patients in the HG had worse OS (20.9 vs. 40.6 months, *p* = 0.035) and PFS (9 vs. 19 months, *p* = 0.022) compared to NG.

In our previous study, longitudinal analysis was hampered by limited re-examinations during follow-up (n = 32). Only some patients were assessed before chemoradiotherapy (n = 15), restricting our ability to evaluate neurotoxicity from radio- and chemotherapy. Additionally, the HG was older, had lower MGMT promoter methylation rates, and a lower KPS than the normosmia group—differences that, though not statistically significant, are crucial in ensuring that the groups were not distributed exactly equally, which led to criticism from readership.

Therefore, this prospective, multicenter study aims to determine whether olfactory dysfunction can serve as an independent prognostic marker for glioblastoma outcomes when controlling for known confounders and following patients longitudinally. The hypothesis is that the disturbance of olfactory function is intrinsically caused by the disease itself and not by external factors such as radiochemotherapy or tumor infiltration in structures of the olfactory tract.

## Methods

This study is a prospective, multicenter observational study. The study was approved by the local Ethics Committee (approval no. 22-10501-BO). In preparing this manuscript, we have used the Strengthening the Reporting of Observational Studies in Epidemiology (STROBE) Statement Checklist as a guide (see supplementary material). The study is conducted in accordance with the Declaration of Helsinki and national regulations.

### Study cohort

The study will enroll 128 participants (64 glioblastoma patients and 64 controls). Glioblastoma patients will be stratified at their first visit after radiotherapy based on extent of resection, MGMT promoter methylation status, initial olfactory test results, MRI findings of olfactory tract involvement, age, and KPS. This approach ensures balanced distribution between normosmia and hyposmia groups. Patients who cannot be assigned to either group will be excluded from further participation. Stratification will occur once post-radiochemotherapy MRI results are available, typically at visit 1 (see Table 1). The controls should essentially match the age and gender of the patients.

**Table 1:** Overview of the planned study visits and their contents

	Screening	Baseline	Visit 1	Visit 2	Visit 3	Visit 4	Follow up visits
Signing Informed consent	x						
Verfication of inclusion and exclusion criteria	x	x					
Stratification			x				
Medical history (including cold symptoms)	x	x	x	x	x	x	x
Physical exam		x	x	x	x	x	x
Karnofsky Performance Status Scale	x	x	x	x	x	x	x
Evaluation of progression and survival data		x	x	x	x	x	x
QoL: EORTC QLQ-C30 and EORTC QLQ-BN20		x	x	x	x	x	x
Recording (severe) adverse events grade III/IV according to CTCAE V5.0		x	x	x	x	x	x
Neurocognitive testing (+ Minimal Mental Status Test)		x	x		x	x	x
Laboratory tests		x	x	x	x	x	x
Olfactory testing	x	x	x	x	x	x	x
MRI	x		x	x	x	x	x
FET PET MRI (if indicated in clinical routine)	x	x	x	x	x	x	x
Laboratory tests for scientific purposes (Biobank)		x	x	x	x	x	x
Next Generation Sequencing (if indicated in clincal routine)	x	x	x	x	x	x	x
Testing for aphasia	x						
Psychosomatic testing	x	x	x	x	x	x	x

### Inclusion and exclusion criteria

The inclusion and exclusion criteria of the study are shown in Table 2.

**Table 2:** Overview of the study's inclusion and exclusion criteria: Pronounced aphasia is defined as severe communication impairment that limits participation in or comprehension of study-related assessments. In cases of suspected speech disorder, a diagnostic evaluation will be performed using the modified Aachen Aphasia Test, the Aachen Aphasia Bedside Test, and the Aphasia Check List. Abbreviations: CNS = central nervous system; WHO = World Health Organization; IDH = isocitrate dehydrogenase; ICU = intensive care unit

Inclusion criteria	Exclusion criteria
At least 18 years of age	Presence of Neurodegenerative diseases (e.g. Parkinson's disease, Alzheimer's disease, Huntington's disease, Korsakoff's syndrome, Pick's disease, Shy-Drager syndrome)
Newly-diagnosed glioblastoma (CNS WHO 2021; IDH wild-type) before start of first radiotherapy	History of invasive tumors or surgery in the head or neck area, except for surgeries for non-invasive skin tumors (e.g. basal cell carcinomas)
Never received prior chemotherapy	Permanent olfactory impairment following infections (e.g., influenza, coronavirus)
Never received radiotherapy to the head or neck before	Conditions that, in the examiner’s judgment, could interfere with the participant’s study compliance (e.g., schizophrenia)
KPS ≥ 70	Language barriers likely to interfere with participation or comprehension of study procedures
No history of severe head or brain trauma requiring ICU admission or classified as Glasgow Coma Scale grade 3	
No respiratory infection at the time of inclusion	
No significant aphasia	

### Study procedures

#### Study visits

Study visits for glioblastoma patients will be scheduled as follows: screening (postoperative, once histology is available), baseline (as soon as MGMT promoter methylation status is determined, but before radiotherapy), visit 1 (4 ± 1 weeks after radiotherapy ends and before adjuvant chemotherapy begins), visit 2 (3 months ± 2 weeks after end of radiotherapy), visit 3 (6 months ± 2 weeks after end of radiotherapy), visit 4 (12 months ± 2 weeks after end of radiotherapy), and follow-up visits (every 6 months ± 2 weeks after visit 4, for a maximum of 2 years).

#### Obtaining informed consent

Informed consent will be obtained during the screening visit.

#### Stratification

Stratification will be performed once during the visit 1 (after radiotherapy), when, among other factors, the patient's MGMT promoter methylation status and group assignment (NG vs. HG) are determined. Additionally, the post-radiotherapy MRI of the skull will be assessed for infiltration of the olfactory tracts. Stratification may result in the exclusion of study participants after visit 1 (see Table 1).

#### Medical history

A detailed medical history—including previous illnesses, surgeries, allergies, intolerances, medication history, autonomic history, gynecological history (for women), history of stimulant use or addiction, and family/social history—will be taken at the screening visit. At each subsequent visit, clinical changes such as new diagnoses, imaging results, therapy adherence, adverse events or serious adverse events (Common Terminology Criteria for Adverse Events (CTCAE) V5.0), tumor progression, new medications, and dosage adjustments will be recorded. Cold symptoms or signs of respiratory infections will be explicitly queried at every visit.

#### Physical examination

A physical examination (neurological and internal) will be performed at each visit, except for the screening visit.

#### KPS scale

The KPS Scale will be assessed at every visit.

#### Disease status (progression)/Survival data

Disease status regarding progression and overall survival will be recorded at each visit.

#### Measuring quality of life

Quality of life will be assessed at every visit except the screening visit using the EORTC QLQ-C30 and EORTC QLQ-BN20.

#### Psychosomatic testing

A structured psychosomatic assessment will be performed. At every visit except the screening visit, participants will complete psychometric questionnaires (Personal Health Questionnaire Depression Scale (PHQ-8), Generalized Anxiety Disorder-7 (GAD-7), National Comprehensive Cancer Network (NCCN) Distress Thermometer, and self-created e-health items) on-site or, if necessary, at home, with assistance from relatives or caregivers allowed. Additionally, a brief qualitative interview on psycho-oncological needs may be conducted with up to ten glioblastoma patients, either in person, by phone, or via secure video call, with each interview lasting no more than 30 min. These interviews are exploratory in nature and serve solely to generate hypotheses regarding the needs and gaps in care for glioblastoma patients. The ten participants are randomly selected.

#### Recording of (serious) adverse events with clinical relevance

Adverse and serious adverse events (based on CTCAE V5.0) will be recorded at each visit except the screening visit. Severity is not the determining factor for documentation; the investigator’s clinical judgment of clinical relevance is key.

#### Investigation of the olfactory sense

The extended Burghart test comprises a threshold and an identification module for detailed olfactory assessment [11]. The threshold test includes 16 dilutions of an odorant (plus blanks), presented in triplets; correct and incorrect choices guide up- or downgrading the dilution level until a consistent threshold is found (score range 1–16). Higher scores indicate a better ability to detect weak odors. The identification test features 16 possible smells, with 12 randomly selected at each test; participants choose the correct smell from four options. Each correct answer scores 1 point (maximum 12). Testing each nostril separately, average scores ≥ 10 classify as normosmia, < 10 as hyposmia.

During screening, only the identification test is performed to assign participants to the normosmia or hyposmia group. At subsequent visits, both threshold and identification modules are done. The threshold test is carried out with both nostrils simultaneously, starting at dilution 16. Participants must avoid eating, drinking, smoking, or chewing gum for at least 15 min before testing. Correct answers are not revealed to maintain test reliability across repeated assessments.

#### Neurocognitive testing

The following neurocognitive tests will be carried out at every visit except the screening visit and visit 2:

- Immediate and delayed story recall from the Rivermead Behavioural Memory Test [3]
- Digit span (forward and backward) and block span (forward and backward) from the Wechsler Memory Scale [8]
- Phonemic verbal fluency from the Regensburg Verbal Fluency Test (phonemic) [2]
- Trail Making Test A and B [14]

#### O-(2-^18^F-fluoroethyl)-l-tyrosine (^18^F-FET)—positron emission tomography (PET) MRI

FET-PET MRI can visualize potential invasive growth in olfactory centers with high-resolution. However, to protect patients, it will only be performed if clinically indicated. FET-PET MRI data will be used in a post-hoc analysis for the study.

#### MRI

All MRIs are used as part of clinical routine at the following points in time: Postoperative (24 h-72 h post surgery), before starting adjuvant chemotherapy (≈4 weeks post-radiotherapy), and approximately 3, 6, 12, 18, and 24 months after radiotherapy. The first post-radiotherapy MRI will be used to determine whether the tumor infiltrates the olfactory tract. A high-resolution coronal T2 sequence is required for this purpose. Two radiologists at independent centers will evaluate all MRIs. In contrast to the local radiologist, the reference radiologist is blinded to the patient's sex, name, and year of birth. Additionally, both radiologists are blinded to each other's assessment regarding olfactory tract infiltration on the MRI. In selected patients, a DTI (diffusion tensor imaging) sequence will be obtained for a more precise assessment of infiltration in olfactory regions.

#### Laboratory tests and blood/tissue sampling for accompanying scientific projects (biobank)

At each visit except screening, approximately 35 mL of blood will be drawn for routine lab tests and for biobanking. Lab values obtained between visits for clinical purposes may also be evaluated for potential therapy-related side effects. In addition, at each visit except screening, a 7.5 mL EDTA sample will be taken and centrifuged, allowing for further analyses (e.g., extracellular vesicle analysis, Western blot or flow cytometry) if needed during the study. Tissue samples that are preserved as part of biobanking can also be used in the course of further research.

#### Next-generation sequencing (NGS) and other molecular analyses

NGS and other molecular analyses of the tumor genome will be conducted when clinically indicated. The locally available methods used in clinical routine (e.g. QIAseq targeted DNA panel, tumor mutation burden (TMB) analysis, the assessment of microsatellite instability status (MSI)) are used for this purpose.

#### Aphasia testing and language barrier assessment

During the screening visit, a speech therapist or study physician will conduct an aphasia and language barrier evaluation if there is clinical suspicion of a speech or language disorder. The goal is to identify communication problems that could impede participation in one or more planned study procedures. Items from the Aachen Aphasia Test, the Aachen Aphasia Bedside Test, and the Aphasia Check List will be used for aphasia testing. Language barriers can be assessed through a brief conversation (e.g., discussing the study content).

#### Sample size calculation and sampling strategy

The sample size was determined for a time-to-event analysis of Overall Survival (OS) using a Cox proportional hazards model. The calculation was based on the primary comparison between glioblastoma patients with hyposmia (defined as'cases'for the calculation) and those with normosmia (defined as'controls'). A hazard ratio (HR) of 0.43 for normosmia vs. hyposmia was used, a value derived from the multivariate analysis for OS in a specific subgroup of patients in the preceding pilot study [12]. As per the pre-specified statistical plan for this prospective trial, event rates over a two-year duration were prospectively defined at 47% for the hyposmia group and 53% for the normosmia group. With a significance level set at 5% and a statistical power of 80%, a total of 128 participants (64 glioblastoma patients and 64 healthy controls) is required to test this hypothesis. To control for key prognostic factors, patients are stratified after enrollment (at Visit 1) based on MGMT promoter methylation, KPS, age, extent of resection and MRI findings of olfactory tract infiltration. These variables, along with olfactory function, will be included as covariates in the final multivariate Cox proportional hazards model to adjust for potential confounding and assess the independent prognostic value of olfaction.

### Statistics

A Cox proportional hazards regression model will be used to describe the event, overall survival, where the variables MGMT, KPS, age, MRI findings of olfactory tract involvement and olfactory function will be used as the covariates. All models will be check for proportionality and the log likelihood will be used as the criteria for model selection. All statistical analysis and graphics will be conducted in R version 3.5.0 (Boston, MA).

## Results

The first participant was enrolled in May 2023. As of April 2025, 32 patients and 9 controls have been recruited. A second study center opened at Münster University Hospital in December 2024, and more centers may be added, aiming to complete enrollment by December 2026. Follow-up will continue until December 2028, with final results of the observational study expected in 2028. Additional translational projects involving biomaterials are planned, and their study protocols are currently under development.

### Perspective

This prospective, multicenter observational study aims to clarify the prognostic significance and underlying mechanisms of olfactory dysfunction in newly diagnosed glioblastoma. Previous retrospective data have suggested an association between impaired olfaction and reduced survival in glioblastoma patients [12], but those findings were limited by potential confounders and lacked longitudinal follow-up. This study was designed to overcome these limitations by integrating rigorous stratification, serial olfactory testing, multimodal imaging, neurocognitive assessments, and molecular profiling.

The primary objective is to determine whether olfactory dysfunction independently predicts overall survival when accounting for established prognostic factors such as MGMT promoter methylation, KPS, age, and extent of resection. In parallel, two mechanistic hypotheses are tested: (1) direct tumor invasion of olfactory-related structures, and (2) neurotoxicity resulting from radiotherapy or chemotherapy. The aim is to prove that an olfactory dysfunction in glioblastoma is intrinsic to the disease and is not caused by radiochemotherapy or local tumor infiltration in the olfactory tract.

To assess structural involvement, high-resolution coronal T2-weighted MRI and DTI are employed to detect infiltration of primary and secondary olfactory regions. Metabolic activity in these areas is further evaluated using FET PET in clinically indicated cases. These imaging data are assessed independently by two neuroradiologists to ensure objective interpretation.

To investigate treatment-related neurotoxicity, olfactory function is longitudinally monitored over two years using both identification and threshold components of the Sniffin’ Sticks test. These data are complemented by repeated neurocognitive testing, and quality of life assessments, allowing for correlation between sensory decline, cognitive function, and psychosocial burden.

The study incorporates matched healthy controls to provide normative data and differentiate disease-specific patterns from age-related olfactory decline. Additionally, tumor genome sequencing and biobanking enable exploratory analyses of molecular correlates of hyposmia and clinical outcome.

Olfactory dysfunction has been implicated in various neurodegenerative disorders and, in oncology, as a marker of treatment-related neurotoxicity in other non-central nervous system malignancies [13, 16, 17]. In glioblastoma, additional mechanisms such as tumor spread via the RMS or microscopic invasion of olfactory structures may contribute [1, 4, 12]. This study is uniquely positioned to disentangle these processes through a multimodal, longitudinal approach.

While the study is ongoing and results are not yet available, its methodological rigor and comprehensive design position it to deliver conclusive insights into the prognostic, diagnostic, and pathophysiological relevance of olfactory dysfunction in glioblastoma. If our hypotheses are confirmed, olfactory testing could emerge as a simple, non-invasive tool to enhance risk stratification, monitor neurotoxicity, and guide personalized care strategies in neuro-oncology.

## Conclusions

This study represents the most comprehensive investigation of olfactory dysfunction in glioblastoma to date, addressing both tumor infiltration and therapy-induced neurotoxicity. Its findings may refine prognostic assessments and inform strategies to mitigate neurotoxic effects, ultimately improving patient outcomes.

## Data Availability

All data generated or analyzed during this study are included in this published article and its supplementary information files.

## Declarations

### Permission to reproduce material from other sources

No material from other sources was used that is not freely accessible.
